# A New Technique for Halving Dental Local Anesthetic Cartridges and Diluting Adrenaline

**DOI:** 10.7759/cureus.87187

**Published:** 2025-07-02

**Authors:** Takutoshi Inoue, Toru Yamamoto, Naotaka Kishimoto

**Affiliations:** 1 Department of Anatomy, Teikyo University School of Medicine, Tokyo, JPN; 2 Division of Dental Anesthesiology, Faculty of Dentistry and Graduate School of Medical and Dental Sciences, Niigata University, Niigata, JPN

**Keywords:** adrenaline, cartridge, coring, dilution technique, intravenous catheter, local dental anesthesia

## Abstract

When using dental local anesthetics containing adrenaline as a vasoconstrictor, it is essential to exercise sufficient caution regarding hemodynamic fluctuations, particularly in patients with cardiovascular disease. In this report, we introduce a safe and simple method to halve the adrenaline concentration in anesthetic cartridges. The equipment used includes a syringe (1-3 mL), an IV catheter (22- or 24-G), and a metal needle, which are typically available in dental clinics for IV sedation or emergency use. By connecting the inner needle (24- or 27-G) of the IV catheter to the syringe and puncturing the edge of the rubber stopper at the head of the cartridge, 0.9 mL of anesthetic solution can be accurately aspirated while reducing the risk of coring. Furthermore, by injecting an equal volume of 2% lidocaine without adrenaline into the cartridge, the adrenaline concentration can be effectively halved. This method is quick, broadly applicable, and does not require any special equipment, making it feasible even for dentists who are not specialists in dental anesthesia. In Japan, four types of dental local anesthetics are commonly used. However, dental treatment cannot always be completed with a preparation that does not contain adrenaline, and there are many situations where the excellent anesthetic and hemostatic effects of adrenaline are required. Dentists must accurately assess these situations and confidently select and administer the appropriate anesthetic. It should be noted that articaine alone is not commercially available in Japan; therefore, this method can only be applied to cartridges containing 2% lidocaine with 1:80,000 adrenaline. Although local anesthetics containing 1:80,000 adrenaline are generally considered safe for patients with cardiovascular disease, the use of the lowest possible adrenaline concentration is desirable. Therefore, this method provides a practical approach to minimize cardiovascular risk while maintaining anesthetic efficacy and hemostasis, especially in procedures requiring large amounts of local anesthetic. Given the increasing number of patients with cardiovascular disease due to aging, this technique is considered a practical approach to ensuring safer dental treatment.

## Introduction

Local anesthesia is a common clinical procedure performed by dental practitioners in daily practice [1]. However, dental local anesthetic cartridges often contain adrenaline, a vasoconstrictor, and a careful approach is required to avoid rapid overdose, particularly in patients with underlying systemic conditions [2].

In Japan, dental local anesthetic (2% lidocaine with 1:80,000 adrenaline) is supplied in dedicated glass cartridges [1]. Due to the relatively high concentration of adrenaline, these formulations may have notable hemodynamic effects, especially in patients with cardiovascular diseases [2].

Previously, we reported two techniques for diluting adrenaline in anesthetic cartridges: one that prevents coring [3] and another that utilizes the inner needle of an IV catheter [1]. These methods allow even general dentists, without specialization in dental anesthesia, to perform simple dilution procedures while also preventing contamination of the solution by rubber fragments caused by coring.

However, to apply these techniques, it is necessary to discard half of the anesthetic solution from the cartridge in advance, during which the risk of coring must also be considered. Therefore, based on our previous findings, we have developed a method to safely extract half the volume of the anesthetic solution from the cartridge. This new step completes a procedural technique that enables the safe dilution of adrenaline within the cartridge, and we herein present the full protocol.

## Technical report

Materials and methods

The materials required for this method include a dental local anesthetic cartridge (2% lidocaine with 1:80,000 adrenaline), a syringe (1-3 mL), an IV catheter (22- or 24-G), and a metal needle.

**Figure 1:**
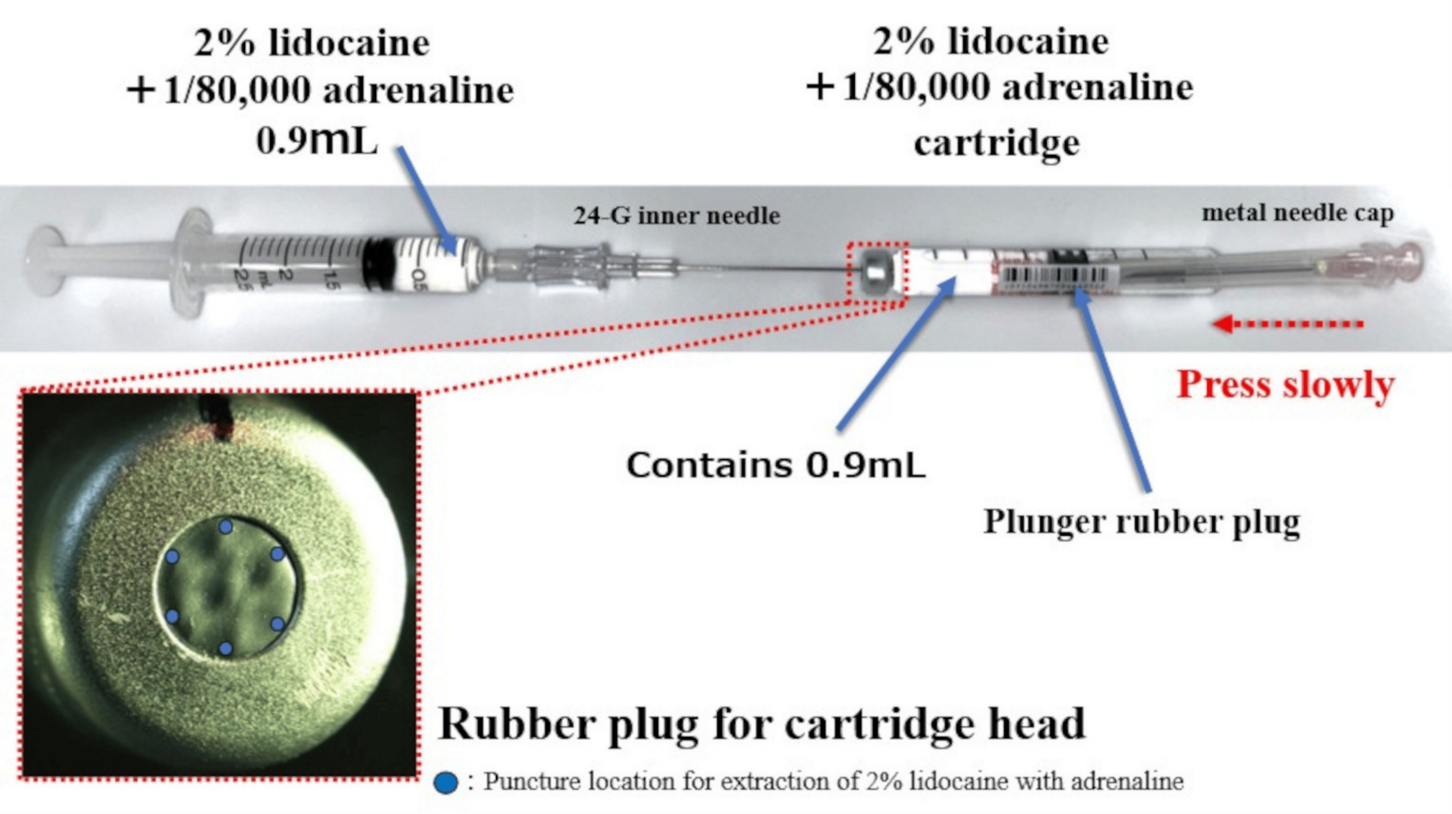
Extraction of local anesthetic from a cartridge with consideration for coring Insert a ≤ 22-G inner needle into the rubber plug end of a dental local anesthetic cartridge (2% lidocaine + 1:80,000 adrenaline), and the rubber plunger at the tail end is gently pressed using the cap of a metal needle to extract the solution. Image credit: Takutoshi Inoue

In our extraction method, (1) a syringe (1-3 mL) is connected to the inner needle (24- or 27-G) of an IV catheter (22- or 24-G) [1]. Due to the inner needle’s length, it should be handled with care. (2) Next, the inner needle is slowly inserted perpendicularly into the edge of the rubber stopper at the head of the cartridge. Since this rubber stopper is typically punctured by a 33-G dental needle (diameter range: 1.50 ± 0.08 mm), using a needle of 22-G or smaller to puncture the peripheral region helps reduce the risk of coring. (3) Finally, the rubber plunger at the tail end of the cartridge is gently pressed using the cap of a metal needle, transferring the anesthetic solution into the syringe. By using a syringe with a scale finer than 0.1 mL, exactly 0.9 mL of solution can be accurately withdrawn. This procedure yields a half-volume (0.9 mL) anesthetic cartridge (Figure 1).

**Figure 2:**
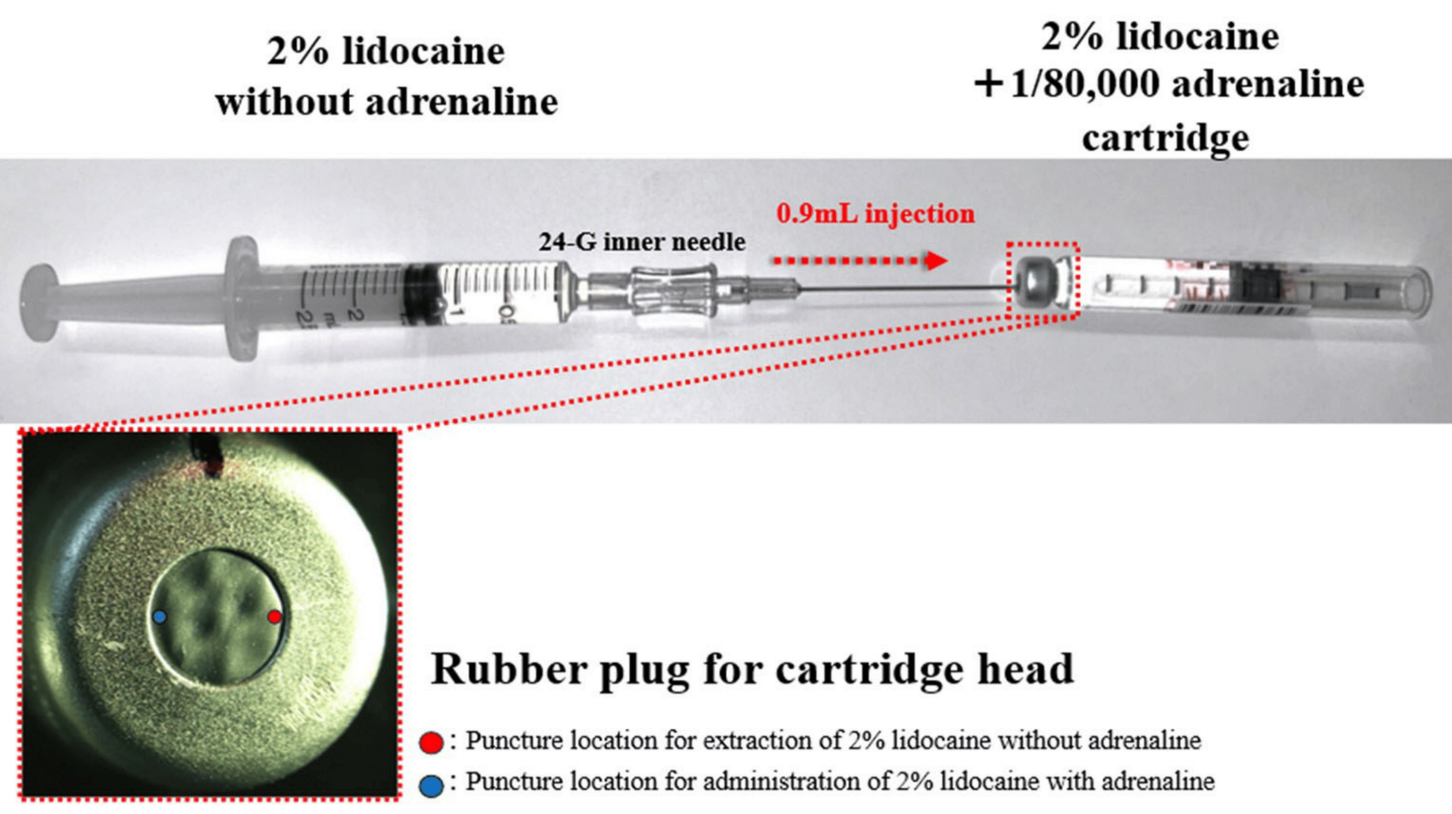
Dilution of adrenaline in a half-volume dental local anesthetic cartridge Insert a ≤ 22-G inner needle into the rubber plug end of a dental local anesthetic cartridge (2% lidocaine + 1:80,000 adrenaline), and 0.9 mL of 2% lidocaine without adrenaline is injected into the cartridge. The injection is performed at a different site than the one used for the previous extraction to reduce the risk of coring. Image credit: Takutoshi Inoue

When using this cartridge to dilute adrenaline, (4) a syringe containing 2% lidocaine without adrenaline that was aspirated is connected to the inner needle [1]. (5) The cartridge head is punctured again at a site different from the initial insertion point used during extraction. (6) Finally, 0.9 mL of 2% lidocaine without adrenaline is then injected into the cartridge [1]. The modified cartridge can then be loaded into a dental syringe or dental electric injector and fitted with a standard dental needle, thus minimizing the risk of coring during administration (Figure 2).

## Discussion

**Figure 3:**
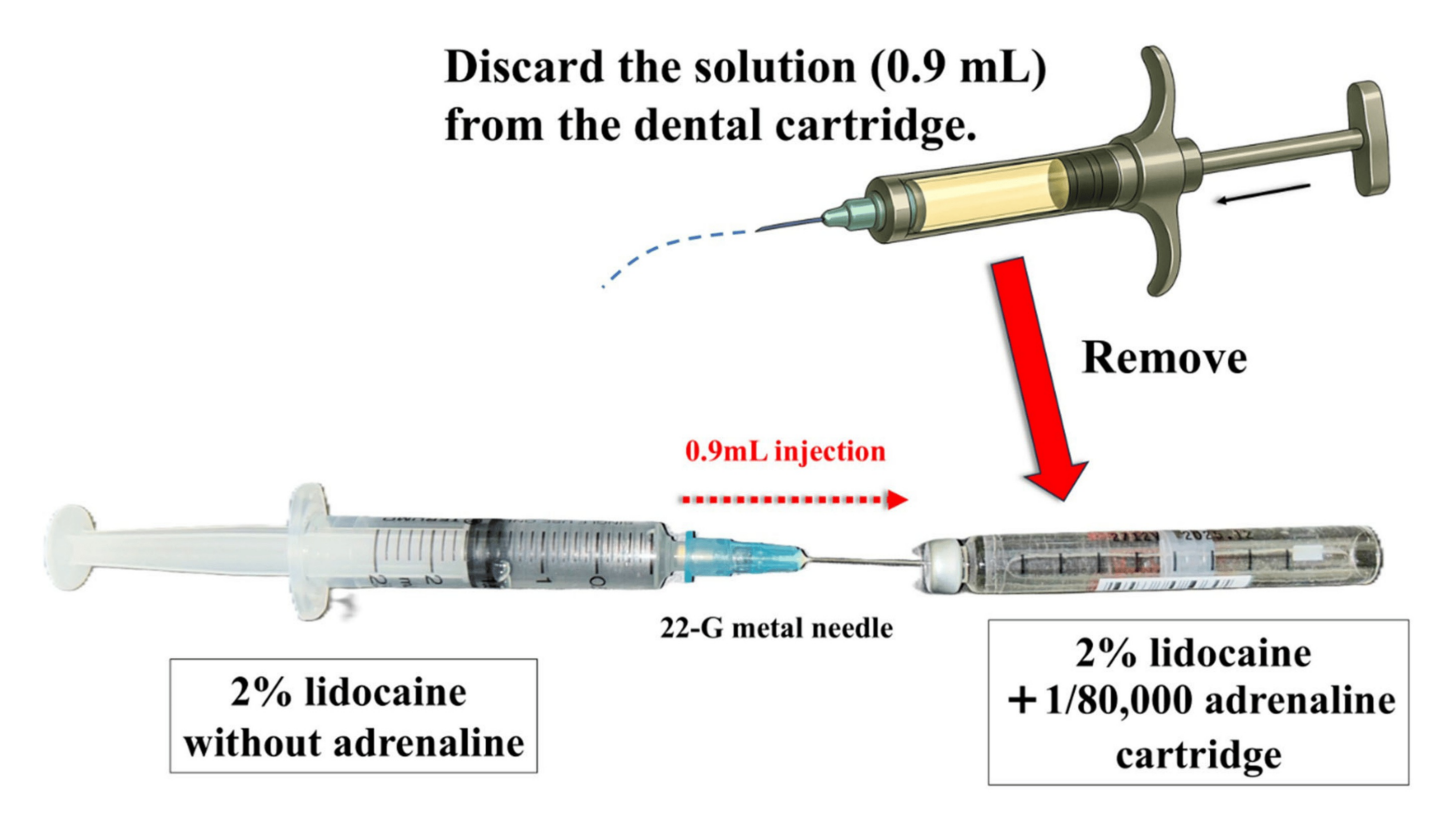
Conventional method for diluting adrenaline in dental local anesthetic cartridges In the conventional method, the cartridge is first loaded into a dental syringe, and the rubber stopper is pierced with a needle to discard half of the solution. Then, 0.9 mL of 2% lidocaine without adrenaline is injected to dilute the remaining adrenaline-containing solution. Finally, the cartridge is reloaded into the dental syringe, and the rubber stopper is pierced again with a dental needle. Image credit: Takutoshi Inoue

In Japan, there has been a longstanding tendency to discard half of the anesthetic solution from cartridges using a dental syringe and dental needle (Figure 3) [4]. However, this method may involve puncturing the same site on the rubber stopper twice with the dental needle, without adequate consideration of the risk of coring [2]. Coring refers to the phenomenon where a portion of the rubber stopper is shaved off by the needle, producing small rubber fragments [3]. Although coring has not been formally reported in the context of dental local anesthetic cartridges, repeated punctures at the same site may increase the risk of rubber fragments contaminating the anesthetic solution [3]. Therefore, analysis of microparticles that contaminate local anesthetic solutions is important from the perspective of ensuring safety.

In recent years, within the field of aesthetic surgery, the extraction of local anesthetic agents from dental anesthetic cartridges has been introduced as a countermeasure to the shortage of local anesthetics [5]. However, this method does not consider the issue of coring that can occur when puncturing the cartridge head.

A distinguishing feature of our proposed method is that it utilizes instruments routinely available in Japanese dental clinics, including syringes, IV catheters, and metal needles. By repurposing tools that are typically stocked for IV sedation or emergency situations, the procedure can be performed without special preparation, which constitutes a significant advantage.

Notably, the anesthetic solution extracted into the syringe using this method can be reused as a local anesthetic for the same patient by attaching an ultra-fine needle (30-33G). However, as these ultra-fine needles are not typically included in standard dental needle sets, they must be purchased separately.

The advantage of reducing the adrenaline concentration in the cartridge is that it may help not only to minimize circulatory fluctuations, but also to maintain anesthetic and hemostatic effects. In fact, local anesthetics containing 1:200,000 adrenaline have been reported to be more effective at stabilizing vital signs than those containing 1:80,000 adrenaline and may be beneficial in hemodynamically unstable patients [6].

Furthermore, the latest systematic review by Guimaraes et al. [7] reported that while the use of local anesthetics containing adrenaline is generally considered safe for patients with cardiovascular disease, previous literature suggests that the lowest effective concentration of adrenaline should be used in such patients whenever possible. From this perspective, although it would be ideal to provide safe dental treatment using adrenaline at a concentration of 1:80,000, the proposed method is considered useful for procedures that require a large volume of local anesthetic.

In Japanese dentistry, there are four types of local anesthetics for dental use (lidocaine + adrenaline, propitocaine + felypressin, mepivacaine + no vasoconstrictors, and articaine + adrenaline) [1,8]. Ideally, safe dental treatment could be provided using local anesthetics that do not contain adrenaline. However, in clinical practice, there are many cases in which the superior anesthetic and hemostatic effects of adrenaline are essential. Dentists are required to accurately judge these situations and select and use local anesthetics with confidence. In such cases, this method may be of help. It should be noted that articaine alone is not commercially available in Japan; therefore, this method can only be applied to cartridges containing 2% lidocaine with 1:80,000 adrenaline.

Japan is experiencing an unprecedented and rapid shift toward a super-aged society [9]. Against the backdrop of an increasing number of patients with cardiovascular diseases, there has been an urgent need to develop a method for diluting existing dental local anesthetic formulations to mitigate hemodynamic fluctuations during administration. The method introduced in this study is both rapid and straightforward, making it practical and immediately implementable in clinical settings.

## Conclusions

Based on previous findings, we present a practical method for accurately extracting half the volume (0.9 mL) of anesthetic solution from a dental local anesthetic cartridge. In this method, the inner needle of an IV catheter is connected to a syringe and slowly inserted toward the edge of the rubber stopper at the cartridge head. The rubber plunger of the cartridge is then gently depressed using the cap of a metal needle, enabling fluid extraction while preventing coring. The use of a syringe with graduations of 0.1 mL allows for precise volume control. This technique utilizes instruments commonly stocked in many Japanese dental clinics for IV sedation or emergency situations, offering both versatility and practical applicability. Although local anesthetics containing 1:80,000 adrenaline are generally considered safe for patients with cardiovascular disease, this technique is useful when even lower adrenaline concentrations are required.
